# Compressive Mechanical Properties and Shock-Induced Reaction Behavior of Zr/PTFE and Ti/PTFE Reactive Materials

**DOI:** 10.3390/ma15196524

**Published:** 2022-09-20

**Authors:** Zhenwei Zhang, Yong He, Yuan He, Lei Guo, Chao Ge, Haifu Wang, Yue Ma, Hongyin Gao, Weixi Tian, Chuanting Wang

**Affiliations:** 1 School of Mechanical Engineering, Nanjing University of Science and Technology, Nanjing 210094, China; 2 State Key Laboratory of Explosion Science and Technology, Beijing Institute of Technology, Beijing 100081, China

**Keywords:** reactive materials (RMs), mechanical properties, constitutive model, quasi-sealed chamber test, shock-induced reaction

## Abstract

Existing research on PTFE-based reactive materials (RMs) mostly focuses on Al/PTFE RMs. To explore further possibilities of formulation, the reactive metal components in the RMs can be replaced. In this paper, Zr/PTFE and Ti/PTFE RMs were prepared by cold isostatic pressing and vacuum sintering. The static and dynamic compressive mechanical properties of Zr/PTFE and Ti/PTFE RMs were investigated at different strain rates. The results show that the introduction of zirconium powder and titanium powder can increase the strength of the material under dynamic loading. Meanwhile, a modified J-C model considering strain and strain rate coupling was proposed. The parameters of the modified J-C model of Zr/PTFE and Ti/PTFE RMs were determined, which can describe and predict plastic flow stress. To characterize the impact-induced reaction behavior of Zr/PTFE and Ti/PTFE RMs, a quasi-sealed test chamber was used to measure the over-pressure induced by the exothermic reaction. The energy release characteristics of both materials were more intense under the higher impact.

## 1. Introduction

Metal/polytetrafluoroethylene (PTFE) reactive materials (RMs) are usually composed of PTFE and single or multiple reactive metal elements. It is a new type of high-efficiency damage material different from traditional energetic materials such as explosives, propellants, and pyrotechnics. Compared with traditional energetic materials, metal/PTFE RMs have high energy density and remain inert under normal conditions [1]. When reactive fragments hit the target, a violent redox reaction occurs under the action of high-velocity impact, releasing a large amount of chemical energy and even deflagration or explosion, which can cause multiple damages to the target [2,3].

Among the existing metal/PTFE RMs, Al/PTFE RMs have attracted extensive attention from scholars. In the 1970s, M. J. Willis firstly reported that the Al/PTFE combination can produce reactions under high-velocity impact. D. B. Nielson and V. S. Joshi et al. [4,5] proposed the preparation method and sintering process curve of Al/PTFE RMs. W. Mock et al. [6] studied the reaction threshold of Al/PTFE RMs by Taylor test. M. N. Raftenberg et al. [7] determined the parameters of the Johnson–Cook constitutive model and pressure-shear damage model of Al/PTFE RMs via the Hopkinson compression bar and universal testing machine, which can describe the dynamic response of the materials under various strain rates. From the perspective of energy release, R. G. Ames [8,9] conducted a secondary impact experiment with a vented chamber device and established the relationship between the pressure change in the quasi-sealed chamber, the impact velocity, and the energy release efficiency combined with theoretical analysis.

In recent years, metal/PTFE RMs have been mainly used in the manufacturing of reactive fragments and reactive liners. However, Al/PTFE RMs have the disadvantages of low density and strength, which restricts their further development. To explore more possibilities of formulation, the reactive metal components in the RMs can be replaced. Both zirconium and titanium have excellent physical and mechanical properties, and their strength and density are higher than aluminum. They are widely used as functional or structural materials in the industry, biomedical, aerospace, and other fields [10,11,12]. It is worth noting that the energy release characteristics of zirconium-based materials and titanium-based materials under impact have also attracted more and more attention, such as W/Zr fragments [13,14], Zr/THV fragments [8] (the energy release is better than Al/PTFE of the same volume), and TNTZ alloys fragments [15]. Therefore, Zr/PTFE and Ti/PTFE RMs have potential applications as energetic materials [16,17]. It is very important to explore the dynamic response and shock response characteristics of Zr/PTFE and Ti/PTFE RMs for the design of their multifunctional properties. The main objectives of the work were to investigate the compressive property under various strain rates and the impact-initiated reaction behavior of Zr/PTFE and Ti/PTFE RMs.

## 2. Materials and Methods

### 2.1. Material Types

In this study, Zr/PTFE and Ti/PTFE RMs with mass ratios of 47.6:52.4 and 32:68 were prepared. The mass ratio is calculated by assuming that Zr and Ti are rapidly oxidized by fluorine in PTFE under impact conditions and the reactions have zero oxygen balance. The reaction chemical equation is as follows:

(1) $${\mathrm{Zr}}_{\left(s\right)}{+(\text{-}C}_{2}F_{4}{\text{-})}_{\left(s\right)}\to {\mathrm{ZrF}}_{4}{}_{\left(s\right)}{+2C}_{\left(s\right)}\;\Delta H=-5769J/g,$$

(2) $${\mathrm{Ti}\;}_{\left(s\right)}{+(\text{-}C}_{2}F_{4}{\text{-})}_{\left(s\right)}\to {\mathrm{TiF}}_{4}{}_{\left(s\right)}{+2C}_{\left(s\right)}\;\Delta H=-5683J/g.$$

The particle size of the Zr powder and Ti powder was 48 μm (Zhuzhou Runfeng New Material Co., Ltd., Zhuzhou, China) and the particle size of the PTFE was 100 μm (DuPont, Wilmington, DE, USA). The purity of Zr, Ti, and PTFE were all above 99%.

### 2.2. Preparation of Specimens

The preparation process of Zr/PTFE and Ti/PTFE RMs refers to the molding and sintering method for preparing PTFE-based RMs [4,5]. The preparation process can be described as follows:

(a) The dried powder of Zr, Ti, and PTFE was filled into a chrome steel tank by mass ratio and ground at room temperature;
(b) The grounded powder was placed into a uniaxially pressed mold, the pressure of 30 MPa was held for 5 min, and a cylindrical sample of Φ10 mm × 10 mm was prepared;
(c) The pressed sample was left at room temperature for 4 h to remove any residual stress. The sample was put into the vacuum sintering furnace for sintering. The sintering temperature control curve has been reported by D. B. Nielson and J. Zhou [1,4].

The theoretical maximum density (TMD) of the sintered Zr/PTFE RMs is 3.21 g/cm^3^, the actual density is 2.97 ± 0.08 g/cm^3^, and the porosity is 7.5%. The TMD of the Ti/PTFE RMs is 2.63 g/cm^3^, the actual density is 2.47 ± 0.03 g/cm^3^, and the porosity is 6.1%.

### 2.3. Mechanical Property Experiment

Quasi-static compression tests were carried out on Zr/PTFE and Ti/PTFE RMs samples (Φ10 mm × 10 mm) at a strain rate of 10^−3^ s^−1^ to 10^−2^ s^−1^, and the moving speed of the cross-head is 0.6 mm/min to 6 mm/min. During the experiment, Vaseline was coated between the specimen and the cross-head to reduce the friction. All experiments were performed at least three times so as to get repeatable results.

The dynamics compression behavior of Zr/PTFE and Ti/PTFE RMs specimens were studied via a split Hopkinson pressure bar (SHPB). The SHPB tests were conducted under 298 K and 373 K to evaluate the role of temperature softening effect. Due to the low wave impedance of PTFE-based RMs, in order to obtain clear transmitted wave signals, aluminum rods were used for the incident rod and transmission rod in the test. The incident rod and transmission rod were both 1500 mm in length, the rod diameter was 14.5 mm, and the bullet length was 300 mm. The sizes of the specimens were Φ10 mm × 10 mm and Φ10 mm × 5 mm. The specimens of Φ10 mm × 5 mm were used for the tests at 373 K to prevent the material from creeping at high temperature. During the test, Φ5 mm × 0.3 mm copper discs were used to adjust the incident pulse and slow down the rising edge of the incident wave. All experiments were performed at least three times so as to get repeatable results. The detailed composition and layout of the SHPB device have been reported by W. Li [18] and R. G. Zhao [19].

### 2.4. Quasi-Sealed Chamber Experiment

**Figure 1:**
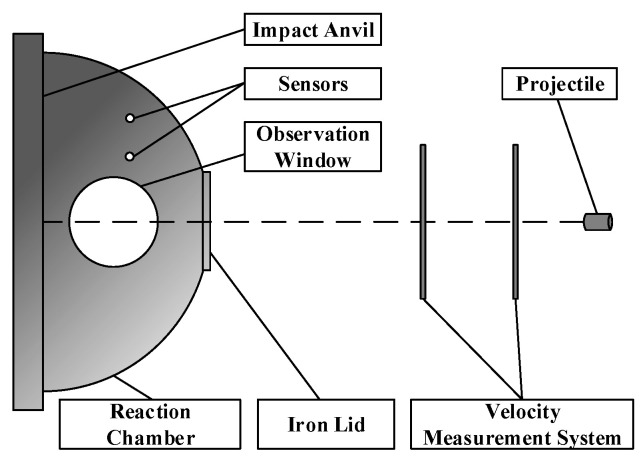
Schematic illustration of the quasi-sealed chamber device.

A hemispherical quasi-sealed chamber was employed in the experiment. This device was improved according to R. G. Ames’ “Vented chamber calorimetry” [9]. The test device is shown in Figure 1. The hemispherical quasi-sealed chamber is made of a thick steel plate with a volume of 23 L, and the front end is sealed with a 0.5 mm thickness iron lid. An observation window and pressure sensors are installed on the outer wall of the chamber to monitor the entire reaction process.

The Zr/PTFE and Ti/PTFE RMs were packed into steel shells to ensure that the materials would not be broken when launching or hitting the iron lid. The size of Zr/PTFE and Ti/PTFE RMs was Φ10 mm × 9 mm, the mass of cylindrical steel shells was 4.6 ± 0.1 g, and the thickness of the steel shells was 1 mm. A ballistic gun was used to launch the projectiles, and the velocity of the projectiles was adjusted by changing the charge quantity. The velocity of the projectiles was measured through the speed measurement system in front of the ballistic gun. The projectiles passed through the iron lid and hit the impact anvil, causing a strong initial impact inside the RMs, triggering a violent chemical reaction and releasing energy.

## 3. Results

### 3.1. Microstructure of the Composites

**Figure 2:**
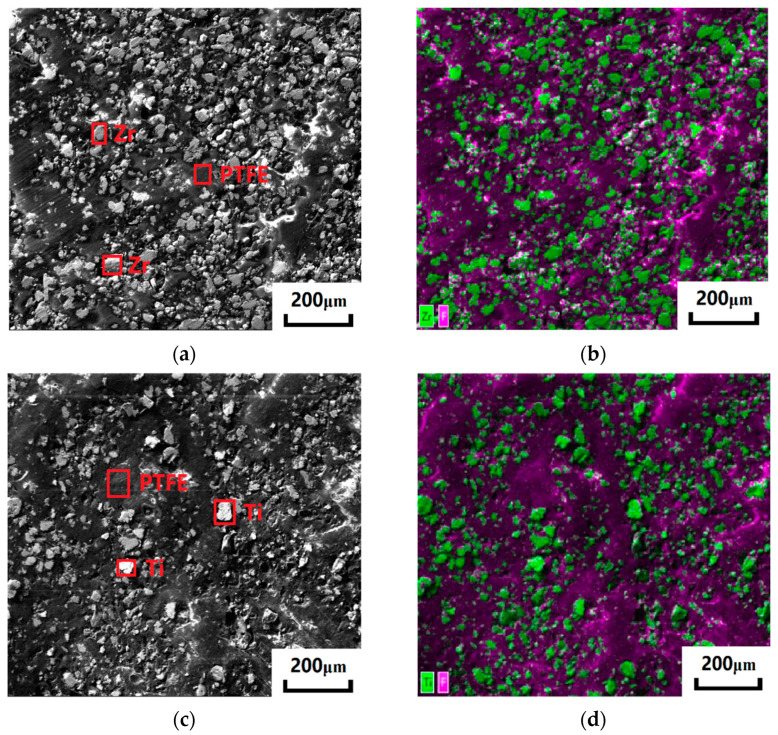
Microstructure of the Zr/PTFE, Ti/PTFE section. (**a**) SEM of Zr/PTFE, (**b**) EDS of Zr/PTFE, (**c**) SEM of Ti/PTFE, (**d**) EDS of Ti/PTFE.

The mechanical property and dynamic responses of sintered materials are closely related to their microstructure. The cross-sections of Zr/PTFE and Ti/PTFE RMs were scanned using a scanning electron microscope (JSM-IT500HR, JEOL Ltd., Tokyo, Japan) under a voltage of 20 kV. It is shown in Figure 2 that the PTFE is dark gray, and the metal particles are bright silver. The sintered PTFE forms a continuous matrix, and the two metal particles are relatively uniformly distributed in the matrix. Both metal particles are irregular in shape, and there are pores between metal particles and PTFE. When the pores are under pressure, cracks will occur due to stress concentration, which will accelerate the failure of the material.

### 3.2. The Mechanical Property under Quasi-Static Compression

**Figure 3:**
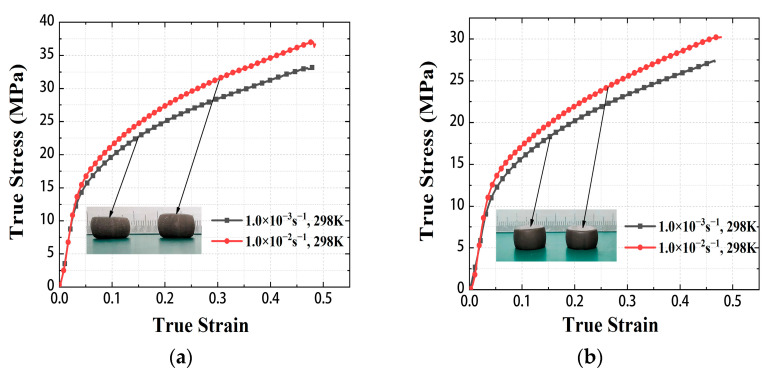
Stress-strain curves of Zr/PTFE, Ti/PTFE under quasi-static compression. (**a**) Zr/PTFE, (**b**) Ti/PTFE.

Deformed specimens and true stress-strain curves of Zr/PTFE and Ti/PTFE RMs under quasi-static compression are shown in Figure 3. The static mechanical properties of Zr/PTFE and Ti/PTFE RMs show obvious plastic properties, with the linear elastic response. After reaching the yield stress, the specimen will produce lateral plastic deformation, and the specimen after compression is in a drum shape. The test results show that when the strain rate is 10^−3^ s^−1^ and 10^−2^ s^−1^, the true stress of Zr/PTFE RMs is 32.1 ± 0.2 MPa and 37.0 ± 0.2 MPa, respectively, while the true stress of Ti/PTFE RMs is 27.4 ± 0.8 MPa and 30.4 ± 0.8 MPa, respectively. Due to the good plasticity of the specimens, an obvious upward setting effect appeared during the compression tests. Therefore, the tests were terminated when the engineering strain reached around 0.4.

### 3.3. The Mechanical Property under Dynamic Compression

**Figure 4:**
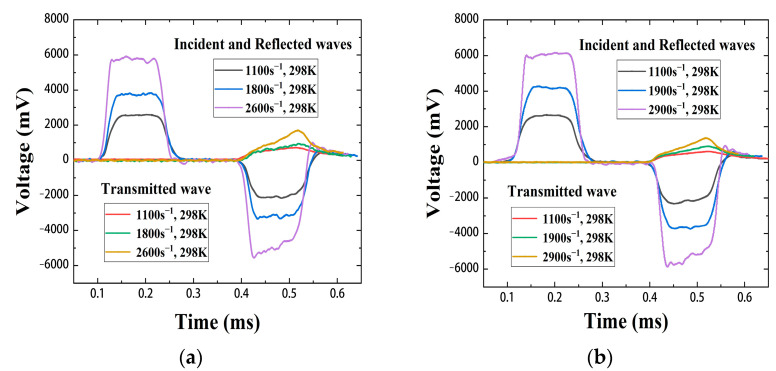
Strain circuit pulse signal-time curves of Zr/PTFE, Ti/PTFE at 298 K. (**a**) Zr/PTFE, (**b**) Ti/PTFE.

During the SHPB test, the high-pressure air chamber drove the bullet to hit the incident rod, and the generated stress wave propagated in the incident rod, the transmission rod, and the specimen. The strain-time signal was collected by the strain gauges on the incident rod and the transmission rod, then adjusted by the Wheatstone bridge to convert it into a voltage pulse-time signal. Figure 4 shows the voltage pulse-time curves under various strain rates at 298 K.

Based on mass conservation and momentum conservation, using the one-dimensional stress wave theory and the assumption of stress uniformity, the true stress σ(t), true strain ε(t), and strain rate $\dot{\varepsilon }(t)$ formulas in the SHPB test can be expressed as follows [20]:

(3) $$\left\{\begin{array}{l} \dot{\varepsilon }(t)=\frac{-2C_{B}}{L_{s}}\varepsilon_{R}(t)\\ \sigma (t)=\frac{E_{B}A_{B}}{A_{S}}\varepsilon_{T}(t)\\ \varepsilon (t)=\frac{2C_{B}}{L_{s}}\int_{0}^{t}\varepsilon_{R}(t)dt \end{array}\right.,$$

where $\varepsilon_{R}(t)$ and $\varepsilon_{T}(t)$ are the reflection and transmission strains generated after the loading pulse, *A_B_* is the cross-sectional area of the compression bar, *A_S_* is the cross-sectional area of the specimen, *C_B_* is the longitudinal propagation velocity of the stress wave in the compression bar, *L_S_* is the length of the specimen, and *E_B_* is the elastic modulus of the compression rod.

**Figure 5:**
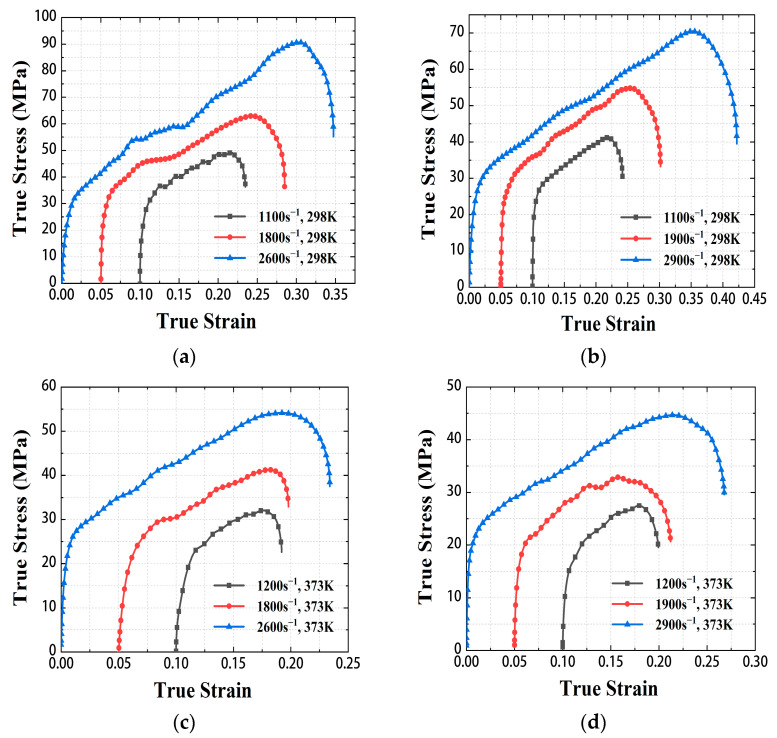
The stress-strain curves of Zr/PTFE, Ti/PTFE under dynamic loading. (**a**) Zr/PTFE, 298 K, (**b**) Ti/PTFE, 298 K, (**c**) Zr/PTFE, 373 K, (**d**) Ti/PTFE, 373 K.

Combined with the pulse signal-time curve of the strain gauge and Equation (3), the true stress and strain curves of Zr/PTFE and Ti/PTFE RMs in the range of strain rate 1100–2900 s^−1^ under 298 K and 373 K were obtained. As shown in Figure 5, the material exhibits typical elastic-plastic mechanical behavior under dynamic loading, and the fluctuation of elastic modulus and yield strength is small in the range of strain rate 1100–2900 s^−1^. In a certain strain range, the stress of the material increases approximately linearly. Meanwhile, both materials showed an obvious temperature softening effect.

According to the true stress-strain curve, the strength of Zr/PTFE is 49 ± 1 MPa, 63 ± 1 MPa, 90 ± 3 MPa at 1100 s^−1^, 1800 s^−1,^ and 2600 s^−1^ strain rates under 298 K, respectively. The strength of Ti/PTFE is 41.3 ± 1 MPa, 55 ± 2 MPa, and 70 ± 1 MPa at 1100 s^−1^, 1900 s^−1^, and 2900 s^−1^ strain rates under 298 K, respectively. Since both Zr/PTFE and Ti/PTFE RMs were fabricated by the same sintering process, the distribution of metal particles in the PTFE matrix was similar. When the metal particle detaches from the PTFE matrix, stress concentrates on the defects in the material, which can lead to material failure [16]. The failure behavior and mechanical properties of the two materials are similar. The Zr/PTFE has slightly higher strength compared to the Ti/PTFE, and the reason may be the different strengths of Zr particles and Ti particles. Under the same conditions, the static mechanical strength of Zr is higher than those of Ti [21]. It is also not excluded that the binding force between the two metal particles and the PTFE matrix might also be different. Similarly, the ultimate strength of the sintered Al/PTFE specimen at a strain rate of about 3000 s^−1^ was 40–62 MPa [18,22], which shows that replacing aluminum with zirconium or titanium improves the strength of the material under dynamic loads.

### 3.4. Shock-Induced Reaction Characteristics

**Figure 6:**
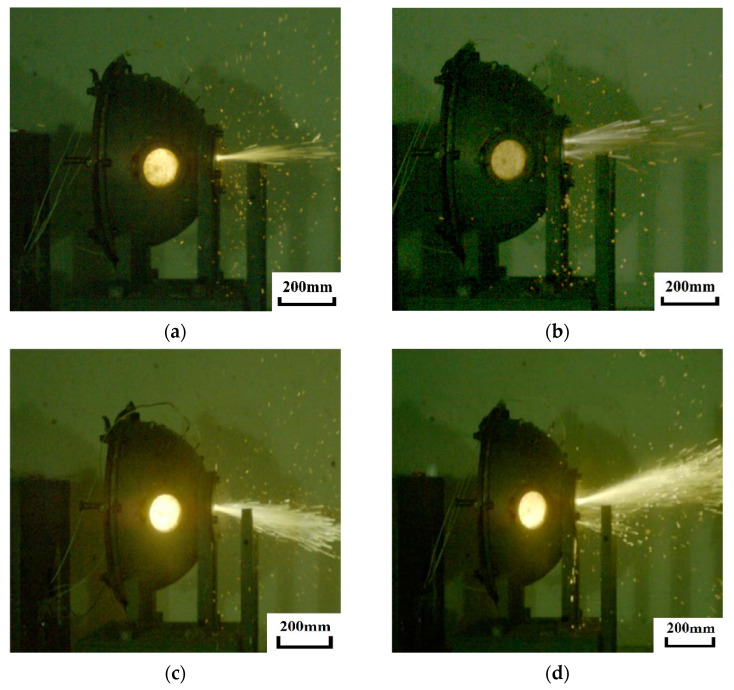
Video frames of Zr/PTFE and Ti/PTFE hitting the impact anvil. (**a**) Zr/PTFE: *v* = 970 m/s, *t* = 11 ms, (**b**) Ti/PTFE: *v* = 990 m/s, *t* = 16 ms, (**c**) Zr/PTFE: *v* = 1250 m/s, *t* = 10 ms, (**d**) Ti/PTFE: *v* = 1340 m/s, *t* = 18 ms.

Figure 6 shows the high-velocity photographic frames of Zr/PTFE and Ti/PTFE RMs impacting the impact anvil at 970–1340 m/s. As shown in the figure, the flash on the observation window is bright, and the gas and reaction products in the chamber are sprayed out from the perforation of the iron lid due to high temperature and high pressure, forming a special venting phenomenon. At the same time, the flash intensity and venting effect increase with the increase of the impact velocity. This shows that both materials undergo a violent chemical reaction under high-velocity impact, and the energy release characteristics will increase with the increase of the impact velocity. The results are consistent with an earlier investigation on Al/PTFE [1,8].

## 4. Discussion

Through the quasi-static compression test and SHPB test, it is shown that Zr/PTFE and Ti/PTFE RMs have obvious plastic properties, and the strength under dynamic loading is higher than that of Al/PTFE RMs. Meanwhile, similar energy release phenomenon to Al/PTFE RMs also occurred when Zr/PTFE and Ti/PTFE RMs impacted the quasi-sealed chamber device under high-velocity.

### 4.1. Validation of SHPB Test Results

**Figure 7:**
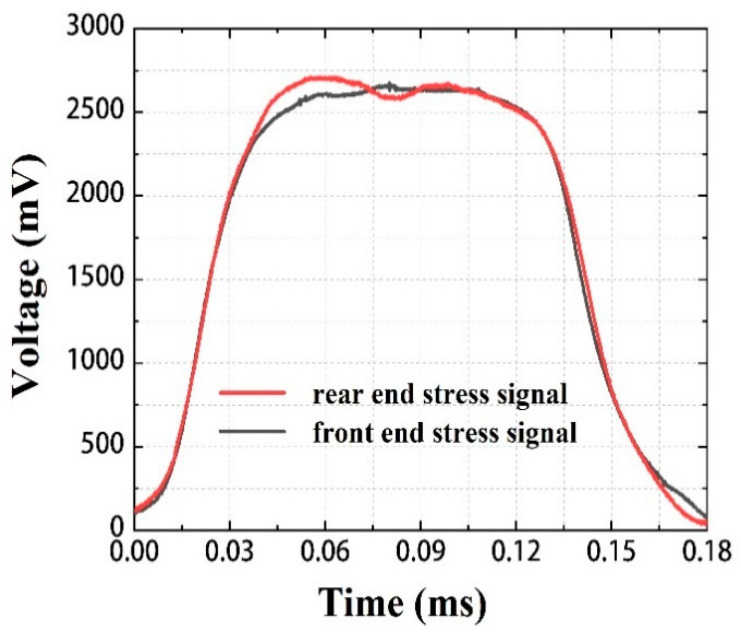
Front and rear stress signals of the specimen under dynamic compression.

In order to ensure the validity of the SHPB test results, it is necessary to ensure that the specimen reaches the dynamic balance of stress—that is, the stress on the front and rear surfaces of the specimen is equal during loading. Since the material of the incident rod and the transmission rod are the same material and have the same diameter, the stress can be directly expressed by the pulse signal of the strain gauge. The front-end stress signal is represented by the absolute value of the incident signal, and the back-end stress signal is represented by the sum of the absolute value of the transmitted signal and the absolute value of the reflected signal. Figure 7 shows the front and rear stress signals of the Ti/PTFE RMs at 1100 s^−1^ strain rates under 298 K. It is shown that the stress signal curve completely coincides with the loading rising stage and the final unloading stage. There is a difference at the peak of the initial load rise, but the stress difference decreases with time. It shows that the specimen reaches dynamic stress balance and deforms at a constant strain rate, and the test results are valid.

### 4.2. Constitutive Model Building

#### 4.2.1. Johnson–Cook Constitutive Model

The constitutive model of the material can describe the mechanical properties and predict the plastic flow stress of the material under various strain rates. The Johnson–Cook model is a purely empirical viscoplastic constitutive model, which has the characteristics of easy fitting of parameters and simple form. It is often used to describe the dynamic mechanical properties of materials under impact loads. Johnson–Cook model considers the strain hardening effect, strain rate effect, and temperature softening effect, and its mathematical form is [23]:

(4) $$\sigma =[A+B\varepsilon^{n}][1+C\ln({\dot{\varepsilon }}^{*})][1-T^{*m}],$$

where σ is the von Mises stress, ε is the equivalent plastic strain,${\dot{\varepsilon }}^{*}=\dot{\varepsilon }/{\dot{\varepsilon }}_{0}$ is the plastic strain rate, $\dot{\varepsilon }$ is the equivalent plastic strain rate, and ${\dot{\varepsilon }}_{0}$ is the reference strain rate of the Johnson–Cook model, generally the quasi-static strain rate of 10^−3^ s^−1^, $T^{*}=\left(T-T_{r}\right)/\left(T_{m}-T_{r}\right)$ is the dimensionless temperature parameter, *T_r_* and *T_m_* are the reference temperature, and the melting point of the material, respectively, and *A*, *B*, *C*, *n*, and m are undetermined parameters: *A* is the yield strength of the material at the reference strain rate and reference temperature, *B* and n are the strain hardening parameters, *C* is the strain rate sensitive parameter, and m is the temperature softening parameter.

#### 4.2.2. Determination of Parameters in Constitutive Models

Since the strain, strain rate, and temperature in the Johnson–Cook model are not coupled with each other, the parameters of the Zr/PTFE RMs constitutive model can be obtained by using the variable separation method and the least-squares method.

Under quasi-static conditions at room temperature, the effects of strain rate strengthening and thermal softening on the flow stress of the materials are ignored, taking the Zr/PTFE RMs as an example: Figure 4 shows that when ${\dot{\varepsilon }}_{0}$ = 1 × 10^−3^ s^−1^, the static yield stress of the Zr/PTFE RMs is 11.596 MPa, that is, *A* = 11.596. So, Equation (4) can be transformed as:

(5) $$\mathrm{lg}\left(\sigma -11.596\right)=\mathrm{lg}B+n\mathrm{lg}\varepsilon .$$

Using the least-squares fitting, *B* = 37.0508, *n* = 0.70609.

The parameter *C* in the Johnson–Cook model can be obtained from the dynamic compression test data at different strain rates under 298 K in Figure 5a [24]. Because the strain hardening effect of the material in the plastic stage has little effect on the flow stress, only the strain rate strengthening effect is considered. Equation (4) can be simplified as:

(6) $$\sigma =11.596(1+C\ln(\dot{\varepsilon }/{\dot{\varepsilon }}_{0})).$$

Fitted using least squares, *C* = 0.11457.

The parameter *m* can be obtained from the dynamic compression test data under 298 K and 373 K in Figure 5a,c. At high temperature and high strain rate, Equation (4) can be simplified as:

(7) $$\sigma (T^{*})=\sigma (T_{r})\left[1-{\left(\frac{T-T_{r}}{T_{m}-T_{r}}\right)}^{m}\right],$$

where $\sigma (T^{*})$ and $\sigma (T_{r})$ represent the yield stress of the material at high temperature (373 K) and the yield stress of the material at the reference temperature (298 K), respectively. *T_r_* is 298 K and *T_m_* is 598 K.

Fitted using least squares, *m* = 0.9536.

In summary, the Johnson–Cook constitutive model of Zr/PTFE RMs is expressed as:

(8) $$\sigma =(11.596+37.0508\varepsilon^{0.7061})(1+0.1146\ln(\dot{\varepsilon }/{\dot{\varepsilon }}_{0}))[1-T^{*0.9536}].$$

Similarly, the Johnson–Cook constitutive model of Ti/PTFE RMs is expressed as:

(9) $$\sigma =(10.227+35.7976\varepsilon^{0.8525})(1+0.1134\ln(\dot{\varepsilon }/{\dot{\varepsilon }}_{0}))[1-T^{*0.937}].$$

#### 4.2.3. Fitting and Modification of Johnson–Cook Constitutive Model

**Figure 8:**
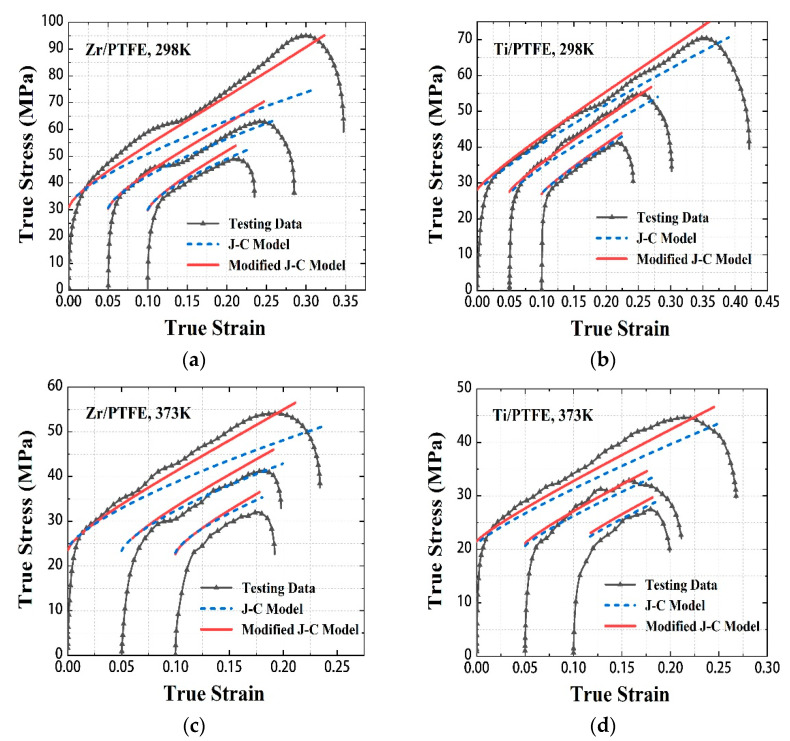
Comparisons of the true stress under dynamic loading for Zr/PTFE, Ti/PTFE between two model predictions and testing data. (**a**) Zr/PTFE, 298 K. (**b**) Ti/PTFE, 298 K. (**c**) Zr/PTFE, 373 K. (**d**) Ti/PTFE, 373 K.

According to the Johnson–Cook constitutive model, the corresponding stress-strain curves are fitted and compared with the existing test curves under dynamic loading. As shown in Figure 8, in the initial plastic stage, the model-predicted curves of the two materials fit well with the experimental curves. However, with the increase of strain rate and strain, the prediction error of the Johnson–Cook constitutive model gradually increases.

According to the research results of Nagy, A [25], the strain and strain rate will have a coupling effect on stress. Therefore, in this paper, a modification of strain and strain rate coupling is added to the Johnson–Cook constitutive model to reduce the error. The modified J-C model is expressed as:

(10) $$\sigma =[A+B\varepsilon^{n}][1+C\ln({\dot{\varepsilon }}^{*})][1-T^{*m}][{\left({\dot{\varepsilon }}^{*}\right)}^{a\varepsilon +b}].$$

The parameter a, b can be obtained from the dynamic compression test data under 298 K and 373 K in Figure 5. The modified Zr/PTFE and Ti/PTFE RMs constitutive models can be expressed as:

(11) $$\mathrm{Zr}/\mathrm{PTFE}\;\mathrm{RMs}:\;\sigma =(11.596+37.0508\varepsilon^{0.7061})(1+0.1146\ln(\dot{\varepsilon }/{\dot{\varepsilon }}_{0}))[1-T^{*0.9536}][{\left(\dot{\varepsilon }/{\dot{\varepsilon }}_{0}\right)}^{0.0485\varepsilon -0.00061}],$$

(12) $$\mathrm{Ti}/\mathrm{PTFE}\;\mathrm{RMs}:\;\sigma =(10.227+35.7976\varepsilon^{0.8525})(1+0.1134\ln(\dot{\varepsilon }/{\dot{\varepsilon }}_{0}))[1-T^{*0.937}][{\left(\dot{\varepsilon }/{\dot{\varepsilon }}_{0}\right)}^{0.0147\varepsilon -0.00157}].$$

As shown in Figure 8, the fitting accuracy of the modified J-C model at high strain rates is improved. At the strain rate of 2600 s^−1^, the fitting errors of Zr/PTFE RMs at 298 K and 373 K decreased from 21.8% to 4.5% and 14% to 4.3%, respectively. At the strain rate of 2900 s^−1^, the fitting errors of Ti/PTFE RMs at 298 K and 373 K decreased from 5.7% to 4.6% and 8.7% to 6.4%, respectively.

#### 4.2.4. Comparison of the Existing Johnson–Cook Constitutive Model

As shown in Table 1, the Johnson–Cook constitutive model parameters of pure PTFE and some PTFE-based RMs are summarized. It is shown that the parameters are close to the range of the parameters of the above constitutive model, which proves the feasibility and universality of the model proposed in this paper.

### 4.3. Shock-Induced Reaction Behavior

**Figure 9:**
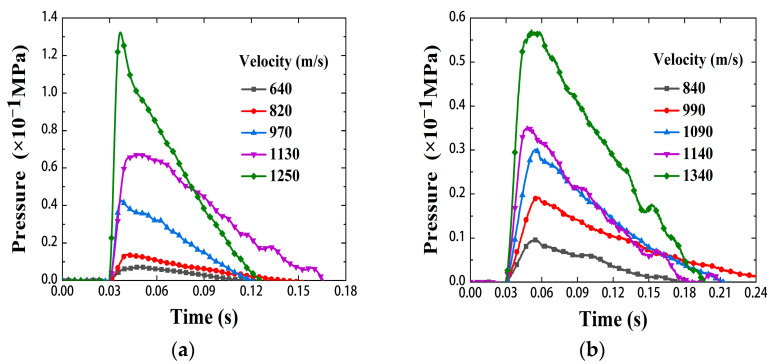
Quasi-static pressure-time curves in the chamber for the Zr/PTFE, Ti/PTFE. (**a**) Zr/PTFE. (**b**) Ti/PTFE.

Figure 9a shows the quasi-static pressure-time curve of Zr/PTFE RMs in the velocity range 640–1250 m/s. Figure 9b shows the quasi-static pressure-time of the Ti/PTFE RMs in the velocity range 840–1340 m/s. It is shown that the pressure in the chamber rises sharply at the initial stage of the reaction, resulting in a pressure peak, and then gradually decreases with the ejection of reaction products and gases. At the same time, the pressure peak will increase with the increase of the impact velocity.

The test chamber can be regarded as a closed system before the pressure peak. The relationship between the quasi-static pressure peak and the energy increase in the chamber can be written as [8]:

(13) $$\Delta P=\frac{\gamma -1}{V}\Delta E,$$

where γ is the air specific heat ratio and the value 1.4. V is the volume of the test chamber. ΔP is the peak pressure value and ΔE is the heat increase of the gas in the chamber, that is, the energy release value of the RMs. Table 2 shows the energy release values of the Zr/PTFE and Ti/PTFE RMs at different velocities. Under the same impact velocity, the release energy of Zr/PTFE RMs is higher than that of Ti/PTFE RMs.

The reaction heat of Zr/PTFE RMs is −5769 J/g and the reaction heat of Ti/PTFE RMs is −5683 J/g. The density of Zr/PTFE RMs is higher than those of Ti/PTFE RMs, so the mass of the Zr/PTFE sample is higher. Besides, the difference in energy release between Zr/PTFE and Ti/PTFE RMs is also closely related to the reaction threshold and the mechanical/chemical properties of the oxide films on the two metal particles [29]. All the above reasons could lead to difference of the reaction intensity between the two composites under impact.

## 5. Conclusions

In this paper, Zr/PTFE and Ti/PTFE RMs were prepared by replacing the metal components in RMs. The compressive mechanical properties of the two materials at different strain rates were studied by quasi-static compression test and SHPB test. In addition, the shock-induced reaction behavior of Zr/PTFE and Ti/PTFE RMs were investigated via the quasi-sealed chamber test. The following conclusions can be drawn:

1. The addition of zirconium and titanium powders improves the strengths of the materials under dynamic load compared to Al/PTFE RMs. The strengths of Zr/PTFE RMs are higher than Ti/PTFE RMs;
2. A modified J-C model considering the strain and strain rate coupling was proposed. The parameters of the modified J-C model of Zr/PTFE and Ti/PTFE RMs were determined, which can describe and predict plastic flow stress;
3. The test results of the quasi-sealed chamber show that the two materials have violent chemical reactions under the high-velocity impact, and the energy release characteristics will increase with the increase of the impact velocity. At the same impact velocity, the released energy of Zr/PTFE RMs is higher than that of Ti/PTFE RMs.

## Figures and Tables

**Table 1:** Summary of the Johnson–Cook constitutive model parameters for PTFE-based RMs.

Material	A	B	n	C	m	a	b
PTFE [26]	11	44	1	0.12	1	/	/
Al/PTFE [7,27] (26.5/73.5)	8.044~17	45.403~250.6	0.86659~1.8	0.4~0.4873	1	/	/
AL/PTFE/W [28] (12/38/50)	23	20.26	0.67604	0.19707	/	/	/
Zr/PTFE (47.6/52.4)	11.596	37.0508	0.7061	0.1146	0.9536	0.0485	0.00061
Ti/PTFE (32/68)	10.227	35.7976	0.8525	0.1134	0.937	0.0147	0.00157

**Table 2:** Calculation results of the energy released by RMs.

Material	Velocity (m/s)	∆*P* (10^−1^ MPa)	∆*E* (J)
Zr/PTFE	640	0.076 ± 0.02	437
	820	0.135 ± 0.03	776.25
	970	0.428 ± 0.03	2461
	1130	0.67 ± 0.05	3852.5
	1250	1.317 ± 0.03	7572.75
Ti/PTFE	840	0.094 ± 0.02	540.5
	990	0.19 ± 0.02	1092.5
	1090	0.29 ± 0.03	1667.5
	1140	0.347 ± 0.03	1995.25
	1340	0.566 ± 0.05	3254.5

## Data Availability

Not applicable.
